# Higher glycolytic capacities in muscle of carnivorous rainbow trout juveniles after high dietary carbohydrate stimulus at first feeding

**DOI:** 10.1186/s12986-019-0408-x

**Published:** 2019-11-09

**Authors:** Yan Song, Hélène Alami-Durante, Sandrine Skiba-Cassy, Lucie Marandel, Stephane Panserat

**Affiliations:** 1 INRA, Univ Pau & Pays de l’Adour, E2S UPPA, UMR1419 Nutrition Metabolism and Aquaculture, Aquapôle, F-64310 Saint-Pée-sur-Nivelle, France; 2 Animal Nutrition Institute, Sichuan Agricultural University, Sichuan, Chengdu, 611130 China

**Keywords:** Fish nutrition, Metabolic programming, Early carbohydrate intake, Early feed restriction, Gene expression, Glucose metabolism

## Abstract

**Background:**

Rainbow trout is a “glucose-intolerant” carnivorous species. Using the metabolic programming strategy, we used early nutritional stimuli in order to modify carbohydrate utilization in trout juveniles.

**Method:**

Fish were fed two diets during the first feeding, namely HP (no carbohydrate / high protein) diet and LP (high carbohydrate / low protein) diet. HP diet was used as the control diet and LP diet as an early stimulus diet. We also used another early stimulus with fish fed HP diet every other day during the first feeding (HP restriction feeding - HPR). After the first-feeding stage (4 weeks), all fish were subsequently subjected to a growth trial with a commercial diet followed by a challenge test with the LP diet (11 weeks). At the end of the first feeding stimulus and of the challenge test, we investigated growth performance, glucose metabolism-related parameters and global DNA C^m^CGG methylation in trout.

**Results:**

LP and HPR dietary stimuli have been a success as shown by the direct modifications of growth performance and mRNA levels for glucose metabolism-related genes at the end of first feeding compared to alevins fed the HP diet. At the end of the challenge trial, no variation in growth performance and hepatic metabolism of LP-history and HPR-history in trout juveniles were observed. However, in muscle of trout juvenile subjected to LP diet at the first feeding, we found an up-regulation of mRNA levels of some glucose metabolism (glucose transport and glycolysis)-related genes and an increase of activities of important glycolysis-related enzymes (hexokinase, phosphofructokinase and pyruvate kinase). These observations are associated with a decrease in the content of glycogen compared to fish fed the HP diet. Moreover, global C^m^CGG DNA methylation in the muscle of fish with LP history was significantly lower than those fed the HP diet.

**Conclusion:**

Dietary LP stimulus at first feeding could permanently modify glucose metabolism and global C^m^CGG DNA methylation level in muscle of trout juveniles, showing that the first feeding stage is efficient for programming the glucose metabolism in fish.

## Introduction

Environmental factors (nutritional or non-nutritional) experienced during the early development can lead to long-term influence on the physiology and metabolism functions of the organism, which is termed metabolic programming or developmental programming [1–5]. In aquaculture, the concept of metabolic programming has attracted broad attention in recent years [6–10]. However, more studies are necessary to better characterize the concept of metabolic programming in fish.

Rainbow trout, a “glucose-intolerant” carnivorous species, showed reduced growth performance and persistent hyperglycemia after intake of high carbohydrate diets [11, 12]. This is the main reason why a new strategy based on metabolic programming have been recently performed in rainbow trout using high dietary carbohydrate at first feeding as an early stimulus to improve their glucose-intolerant phenotype and its use of dietary carbohydrates in juveniles [13, 14]. Indeed, previous studies in rainbow trout demonstrated that a huge hyperglucidic stimulus (60% of dietary carbohydrates and 20% of crude protein) during 3 up to 5 days at the first feeding was able to permanently improve carbohydrate digestive capacity but also, unexpectedly, decrease the muscle glucose transport and glycolysis at the juvenile stage [13, 14]. By contrast, using other fish species i.e. the gilthead seabream, early high-glucose stimulus during a longer period of rearing could improve ^14^C starch utilization in seabream juveniles [15]. These data illustrate that differences in metabolic programming could appear that can be due to differences either in species-related responses (feeding habit, early development and rearing temperature) or to the early feeding protocol (composition and duration). In the present study, we decided to use a HP diet (containing 0% of carbohydrate – 60% of protein as described by Geurden et al. [14]) and a new LP diet (for the first time) as two stimuli during a longer period (4 weeks) to test the hypothesis of improving carbohydrate utilization in trout juveniles through nutritional programming. For the new LP diet, dietary protein content was designed to be near 40% (the intermediate value of protein contents in hyperglucidic diet (20%) and HP diet (60%) as reported by Geurden et al. [14]), which could satisfy the protein requirement of trout alevins [16]), with inclusion of 30% of dietary starch.

Dietary energy restriction applied during early developmental stage could also induce long-term metabolic changes of individuals later in life [4]. Several studies in mammals showed that prenatal and neonatal dietary energy restriction can affect metabolism and physiology in the offspring. In rat, maternal energy restriction to 50 % of ad libitum intake during the last week of pregnancy impaired β-cell development of offspring; continued maternal energy restriction during lactation caused a long-term reduction in β-cell mass and number, and resulted in glucose intolerance in the offspring [17, 18]. In goat weaned progenies, dietary energy restriction during early life have long-term detrimental effects on morphological development of rumen and small intestine [19]. In their natural environment, trout alevins (fries) at the first feeding stage may face a poor-nutrient environment due to the decrease of natural preys (insects) and they can resist up to 9 days of fasting [20]. Up to date, no study has been performed to test the existence of a metabolic programming in juvenile fish induced by early dietary energy restriction (feed restriction). Because it has been reported that feed restriction in fish could reduce production cost and minimize negative effect on environment in aquaculture [21], it is worth now to investigate if a dietary energy restriction (feed restriction) could be linked to metabolic programming in fish in aquaculture. This is the second question of the present study.

Thus, the main objectives of the present study were to investigate the existence of a glucose metabolic programming linked to two early stimuli at the first feeding either with an early dietary carbohydrates intake or with a dietary energy restriction. The analysis of the metabolic programming will be assessed by measuring the growth performance, whole body composition, plasmatic metabolites and glucose metabolism in liver and muscle, two key tissues involved in glucose use. Indeed, as the liver is the center of the intermediary metabolism [11], muscle is the largest part of the fish weight and considered as a stronger user of glucose as a source of energy than other tissues [22, 23]. Moreover, remodeling of epigenetic landscapes is an important mechanism mediating persistent metabolic programming strategy [24]. Nutritional status can affect the way of gene transcription therefore biological processes through epigenetic alterations, such as global DNA methylation [25], which has been also found recently in rainbow trout [8, 26]. This is the main reason why we have measured also the global C^m^CGG DNA methylation in trout juveniles linked to the two early stimuli.

## Material and methods

### Ethical issues and approval

The experiments were conducted according to French and European legislation for the use and care of laboratory animals (Décret 2001–464, 29 May 2001 and Directive 2010/63/EU, respectively). This protocol and the project as a whole were approved by the French National Consultative Ethics Committee and the “Ministère de la Recherche et de l’Innovation”, number APAFIS#10803-2017071017221313v4.

### Diet, fish and experimental design

Two experimental extruded diets for trout alevins, namely HP diet for no carbohydrate / high protein diet (the control diet) and LP diet for high carbohydrate / low protein diet, were prepared at INRA, Donzacq, France (Table 1). Fish meal was included as the unique protein source, gelatinized corn starch was used as the carbohydrate source, whereas dietary lipids were provided by fish oil and fish meal.

**Table 1:** Formulation and proximate composition of the two experimental diets

Ingredients g/100 g diet	HP	LP
Fish meal^a^	94.0	52.0
Fish oil^b^	2.0	14.0
Starch^c^	0.0	30.0
Vitamin mix^d^	1.0	1.0
Mineral mix^e^	1.0	1.0
Alginate	2.0	2.0
Proximate composition		
Dry matter (DM,% diet)	94.58	91.50
Crude protein (% DM)	69.25	41.77
Crude lipid (% DM)	9.51	16.27
Gross energy (kJ g^− 1^ DM)	20.82	22.59
Ash (% DM)	18.18	10.82
Starch (% DM)	0.31	23.79

HP, high protein diet; LP, low protein diet.^a^ Sopropeche, Boulogne-sur-Mer, France; ^b^ North Sea fish oil, France, Sopropeche; ^c^ Gelatinized corn starch (Roquette, Lestrem, France); ^d^ Suppplied the following (kg^−1^ diet): DL-α-tocopherol acetate 60 IU, sodium menadione bisulphate 5 mg, retinyl acetate 15,000 IU, cholecalciferol 3000 IU, thiamine 15 mg, riboflavin 30 mg, pyridoxine 15 mg, vitamin B_12_ 0.05 mg, nicotinic acid 175 mg, folic acid 500 mg, inositol 1000 mg, biotin 2.5 mg, calcium panthothenate 50 mg, choline chloride 2000 mg. ^e^ supplying the following (kg^− 1^diet): calcium carbonate (40% Ca) 2.15 g, magnesium oxide (60% Mg) 1.24 g, ferric citrate 0.2 g, potassium iodide (75% I) 0.4 mg zinc sulphate (36% Zn), 0.4 g, copper sulphate (25% Cu) 0.3 g, manganese sulphate (33% Mn) 0.3 g, dibasic calcium phosphate (20% Ca, 18% P) 5 g, cobalt sulphate 2 mg, sodium selenite (30% Se) 3 mg, potassium chloride 0.9 g and sodium chloride 0.4 g

**Fig. 1:**
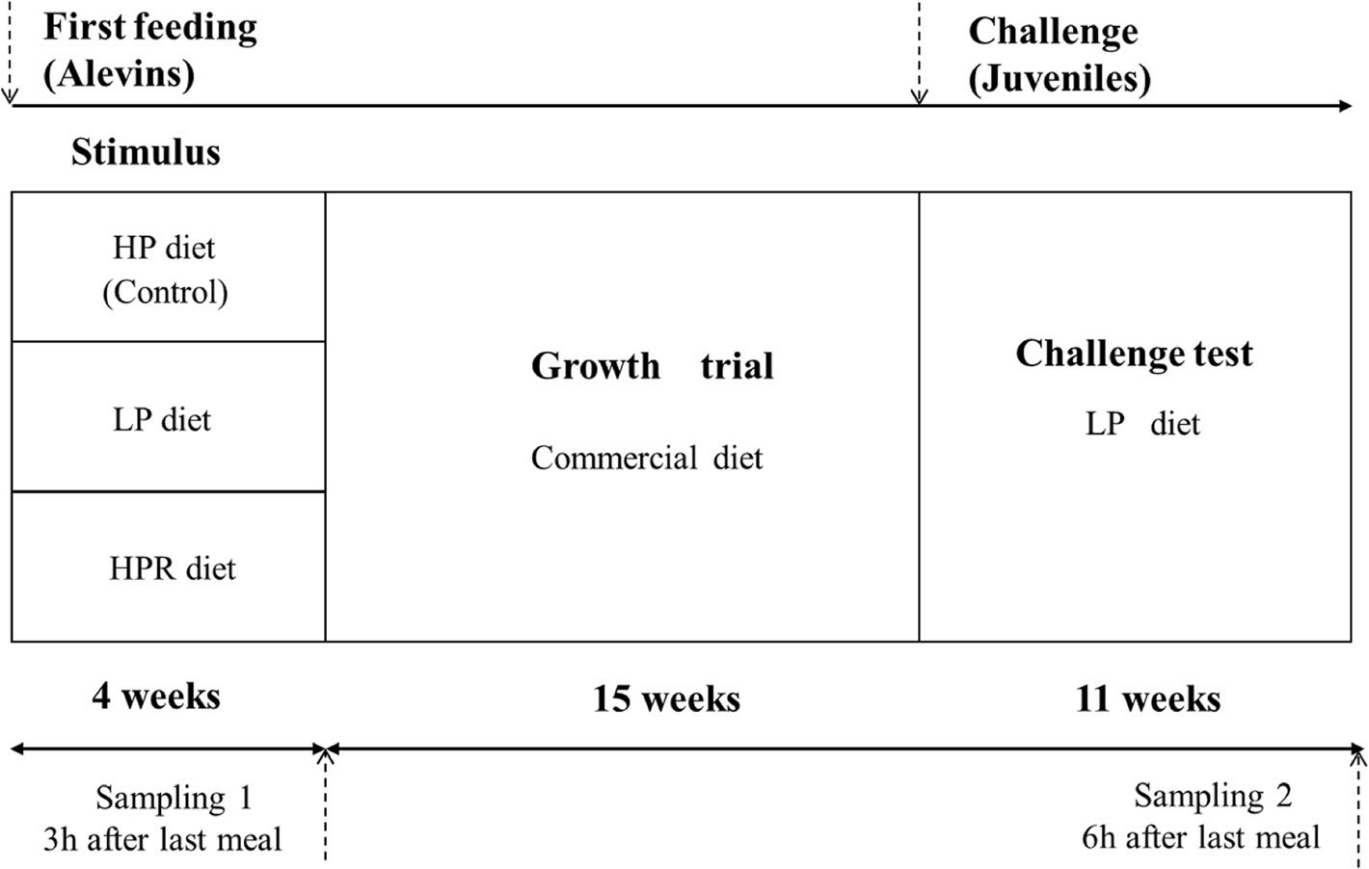
Experimental design. LP and HPR dietary stimulus were applied to rainbow trout alevins for 4 weeks at the first feeding, then the fish were fed the commercial diet. After a growth trial of 15 weeks, fish were subjected to a 11 weeks challenge test with LP diet. HP diet: without carbohydrates. LP diet: with carbohydrates. HPR: fish fed HP diet every other day

The experimental design is detailed in Fig. 1. Rainbow trout eggs were obtained from Lees Athas, INRA fish farm facilities, France. After hatching, trout alevins (initial average weight is 0.11 g) were randomly distributed into experimental tanks with the density of 140 fish per tank in the Donzacq experimental farm. For the early nutritional stimulus (at first feeding), trout alevins were fed with HP or LP diets during 4 weeks. Trout alevins called HPR (dietary energy restriction group) were fed with the HP diet every other day during 4 weeks also (1 day fed, 1 day unfed, 1 day fed, etc.…). Each treatment was performed in triplicate (*n* = 9 tanks in total). Fish were fed eight times daily for 4 weeks and the uneaten feed was collected after feeding. During the dietary stimulus at first feeding, the mortality of alevins was recorded every day in order to calculate the survival rate. After the early nutritional stimulus, all fish groups were fed with a commercial diet (Skretting; 62–58% proteins, 16–18% lipids, 9–12% of carbohydrates) during a period of 15 weeks (called in the present study the “growth trial”). Fish were fed twice a day. The residual feed pellets were collected for correcting the feed intake. At the end of the growth trial, juvenile fish were subjected to a dietary challenge for 11-weeks with LP diet in order to test the existence of a metabolic programming due to the early feeding (“challenge trial”). As for the growth trial, fish were fed twice a day. The residual feed pellets were also collected for correcting the feed intake. During the periods of nutritional stimulus, growth trial and challenge test, fish were reared in a flow-through rearing system supplied with natural spring water (18 °C) under a natural photoperiod, and water quality was checked every 2 weeks.

### Samplings and nutritional parameters

At the end of the nutritional stimulus, three fish per tank were sampled 3 h after the last meal (Fig. 1, Sampling 1). Fish (whole body) were anaesthetized in a benzocaine bath at 30 mg·L^− 1^ and then killed in a benzocaine bath at 90 mg·L^− 1^ 3 h after the last meal (postprandial peak of nutrition absorption for alevins). Then the samples were stored at − 80 °C until analyses. At the end of the challenge trial, a second sampling was performed 6 h after the last meal (Fig. 1, sampling 2). Three fish per tank were euthanized 6 h after the last meal (postprandial peak of nutrition absorption for juveniles) using the same method. Blood was collected from the caudal vein using heparinized syringes and then centrifuged (3000 g, 4 °C, 5 min) to get the plasma. The obtained plasma was immediately frozen and stored at − 20 °C until using. Liver and white muscle (sampled under the dorsal fin) were dissected and immediately frozen in liquid nitrogen, then stored at − 80 °C pending analyses. Subsequently, three fish per tank were randomly sampled 48 h after the last meal, and then immediately frozen at − 20 °C for whole body composition measurement.

The parameters for growth performance, such as survival, specific growth rate (SGR), feed intake (FI) and feed efficiency (FE), were measured every 3 weeks and calculated as follow: (1) Survival (%) = 100 × final fish number/initial fish number; (2) Specific growth rate (SGR, %d^− 1^) = 100× [Ln (final average wet body mass)–Ln (initial average wet body mass)]/d; (3) Feed intake (%/d) = 100 × {dry feed intake/[(initial wet body mass + final wet body mass)/2]}/d; (4) Feed efficiency (FE) = (the mass for dead fish + final wet body mass -initial wet body mass)/dry feed intake, Where d is the experimental period in days.

### Analysis methods: metabolites and enzymatic activities

The chemical composition of diets and whole body composition were measured as previously described by Song et al. [12]. Plasma glucose, triglycerides, lactate and free fatty acids were analysed with Glucose RTU (BioMerieux), PAP 150 (Biomerieux), Lactate PAP (Biomerieux) and NEFA C (Fuji Chemicals GmbH) kits, respectively, according to the recommendations of each manufacturer (*n* = 9 samples per experimental treatment). Total plasma free amino acid concentrations were determined by the ninhydrin reaction according to the method of Moore with glycine as standard [27]. Muscle hexokinase (HK) and phosphofructokinase (PFK) activities were measured according to Borges et al. [28] (*n* = 9 samples per experimental treatment). Muscle pyruvate kinase (PK) activity was determined as previously described by Panserat et al. [29] (n = 9 samples per experimental treatment). Liver and muscle glycogen levels were measured by a hydrolysis technique as reported by Good et al. [30] (*n* = 9 samples per experimental treatment). Liver and muscle glycogen was determined by a hydrolysis technique previously described by Good et al. [30] and Song et al. [12] (*n* = 6 samples per experimental treatment).

### qRT-PCR analysis

The analysis of mRNA levels was performed in trout alevins as well as liver and muscle of trout juveniles (*n* = 9 samples per experimental treatment). Samples were homogenized in Trizol reagent (Invitrogen, Carlsbad, CA, USA) with Precellys®24 (Bertin Technologies, Montigny-le-Bretonneux, France), and then total RNA was extracted following the manufacturer instructions. Total RNA (1 μg) was synthesized to cDNA in duplicate using the SuperScript III RNase H-Reverse Transcriptase kit (Invitrogen) with random primers (Promega, Charbonniéres, France). The primer sequences applied in quantitative real-time PCR (qPCR) assays for selected genes are shown in Table 2. The real-time RT-PCR assays were conducted according to Liu et al. [31]. *Luciferase* and *ef1α* genes were used as reference genes for normalization (luciferase for alevins, ef1α for liver and muscle) of mRNA levels of target genes in alevins and juvenile (liver and muscle) respectively through the E-method on Light Cycler software according to Panserat et al. [8] and Borges et al. [28].

**Table 2:** List of the primers used for qRT-PCR to analyze the expressions of genes involved in glucose metabolism

Genes	Forward primers	Reverse primers
*luciferase*	5′-CATTCTTCGCCAAAAGCACTCTG-3′	5-AGCCCATATCCTTGTCGTATCCC-3′
*ef1α*	5′-TCCTCTTGGTCGTTTCGCTG-3′	5-ACCCGAGGGACATCCTGTG-3′
*glut2a*	5′-GACAGGCACTCTAACCCTAG-3′	5′CTTCCTGCGTCTCTGTACTG-3′
*glut2b*	5′-CTATCAGAGAACGGTACAGGG-3′	5′CAGGAAGGATGACACCACG-3′
*glut4a*	5′-CATCTTTGCAGTGCTCCTTG-3′	5′CAGCTCTGTACTCTGCTTGC-3′
*glut4b*	5′-TCGGCTTTGGCTTCCAATATG-3′	5′GTTTGCTGAAGGTGTTGGAG-3′
*hk1*	5′-CTGGGACGCTGAAGACCAGA-3′	5′-CGGTGCTGCATACCTCCTTG-3′
*hk2*	5′-GGGACACCGAGAACAAGGG-3′	5′-TCCCTTTGTCATCCTGTGCT-3′
*pkmaa*	5′-ACATTGCCCCCTACAGTTAC-3′	5′-AAGTGGAAATGAATGGGACGT-3′
*pkmab*	5′-TGCTGAGGGCAGTGACGTA-3′	5′-AGCTCCTCAAACAGCTGTCTG-3′
*pkmba*	5′-CAAGCCTGCCAACGATGTC-3′	5′-CAAGGAACAAGCACAACACG-3′
*pkmbb*	5′-CAACTGTGACGAGAAGCACC-3′	5′-GAGCCCAGAGTACCACCATT-3′
*gcka*	5′-CTGCCCACCTACGTCTGT-3′	5′-GTCATGGCGTCCTCAGAGAT-3′
*gckb*	5′-TCTGTGCTAGAGACAGCCC-3′	5′-CATTTTGACGCTGGACTCCT-3′
*pfkla*	5′-GATCCCTGCCACCATCAGTA-3′	5′-GTAACCACAGTAGCCTCCCA-3′
*pfklb*	5′-AGTGCTCGCTGTAAGGTCTT-3′	5′-GTGATCCGGCCTTTCTGAAC-3′
*pklr*	5′-CCATCGTCGCGGTAACAAGA-3′	5′-GCCCCTGGCCTTTCCTATGT-3′
*pfkmaa*	5′-GTCAGTCTGTCCGGTAACCA-3′	5′-ATCTGGAGGGTTGATGTGGG-3′
*pfkmab*	5′-TCAGCGGAGGAGGCTAATC-3′	5′-GACTCTGTGCAGTAGTCGTG-3′
*pfkmba*	5′-CTGGGCATGAAAAGGCGAT-3′	5′-GTCTTCTTGATGATGTGCTCCA-3′
*pfkmbb*	5′-CGGTCGTATCTTTGCCAACATG-3′	5′-TGTCCATTTCCACAGTGTCATATT-3′
*pck1*	5′-ACAGGGTGAGGCAGATGTAGG −3′	5′-CTAGTCTGTGGAGGTCTAAGGGC −3′
*pck2*	5′-ACAATGAGATGATGTGACTGCA-3′	5′-TGCTCCATCACCTACAACCT-3′
*fbp1b1*	5′-CTCTCAAGAACCTCTACAGCCT-3′	5′-TCAGTTCTCCCGTTCCCTTC-3′
*fbp1b2*	5′-ATCAGCAGGAATAGGTCGCG-3′	5′-CCTCCTCCAGCACGAATCTC-3′
*fbp1a*	5′-GACAGAGGACGACCCGTG-3′	5′-GTACTGACCGGGTCCAACAT −3′
*g6pca*	5′- GATGGCTTGACGTTCTCCT-3′	5′- AGATCCAGGAGAGTCCTCC-3′
*g6pcb1*	5′-AGGGACAGTTCGAAAATGGAG-3′	5′-CCAGAGAGGGAAGAAGATGAAGA-3′
*g6pcb2*	5′-CCTGCGGAACACCTTCTTTG-3′	5′-TCAATTTGTGGCGCTGATGAG-3′
*ldhaa*	5′-GTGTTTCTCAGCGTTCCCTG-3′	5′-GTTACAGAAGGGCACACAG-3′
*ldhab*	5′-GTGTTCCTCAGTGTGCCATG-3′	5′-TTGCTGATAAATTAACCCTCCG-3′
*slc16a3a*	5′-TAGTGATGTCAAGGCACCAGAT-3′	5′-CACTCCGAACTCCCTGATCAAC-3′
*slc16a3b*	5′-GAGTTGCAGGCTGTAGACC-3′	5′-GCTCACCACAAACACAGGG-3′

*Ef1α: elongation factor 1 alpha; glut***,** *glucose transporter; hk, hexokinase; pkm, pyruvate kinase (muscle); gck, glucokinase; pfkl,phosphofructokinase (liver); pklr, pyruvate kinase (liver and red blood cell); pfkm, phosphofructokinase (muscle); pck, phosphoenol pyruvate carboxykinases (cytosolic pck1 and mitochondrial pck2); fbp, fructose 1, 6-bisphosphatase; g6pc, glucose 6-phosphatase; ldha,Lactate dehydrogenase A; Slc16a3, solute carrier family 16 (monocarboxylic acid transporters) member 3*

### DNA extraction and global DNA CpG methylation analysis

Genomic DNA extraction was performed on the muscle (juvenile fish) of nine fish per experimental treatment as previously described by Liu et al. [26]. DNA quality and quantity were assessed using 1% agarose gel and Qubit dsDNA HS assay kit, respectively. Global DNA C^m^CGG methylation pattern was determined using the method of luminometric methylation assay (LUMA) according to Karimi et al. [32]. Each analysis was carried out in duplicate with 2.5 μg muscle DNA samples according to the manufacturer’s instructions.

### Statistical analysis

The effects of nutritional stimulus (HP, LP and HPR diets) at the first feeding on the different parameters were tested using one way ANOVA test (statistical R software/R Commander package). For statistical analyses, differences were considered significant at *p* < 0.05. Normality of distributions was assessed using the Shapiro-Wilk test whereas the homeostatiscity of variance was evaluated using the Brown-Forsyth test. The results are presented as mean ± SD (standard deviation).

## Results

### Survival and growth performance of rainbow trout from early stimulus up to the end of the dietary challenge

**Fig. 2:**
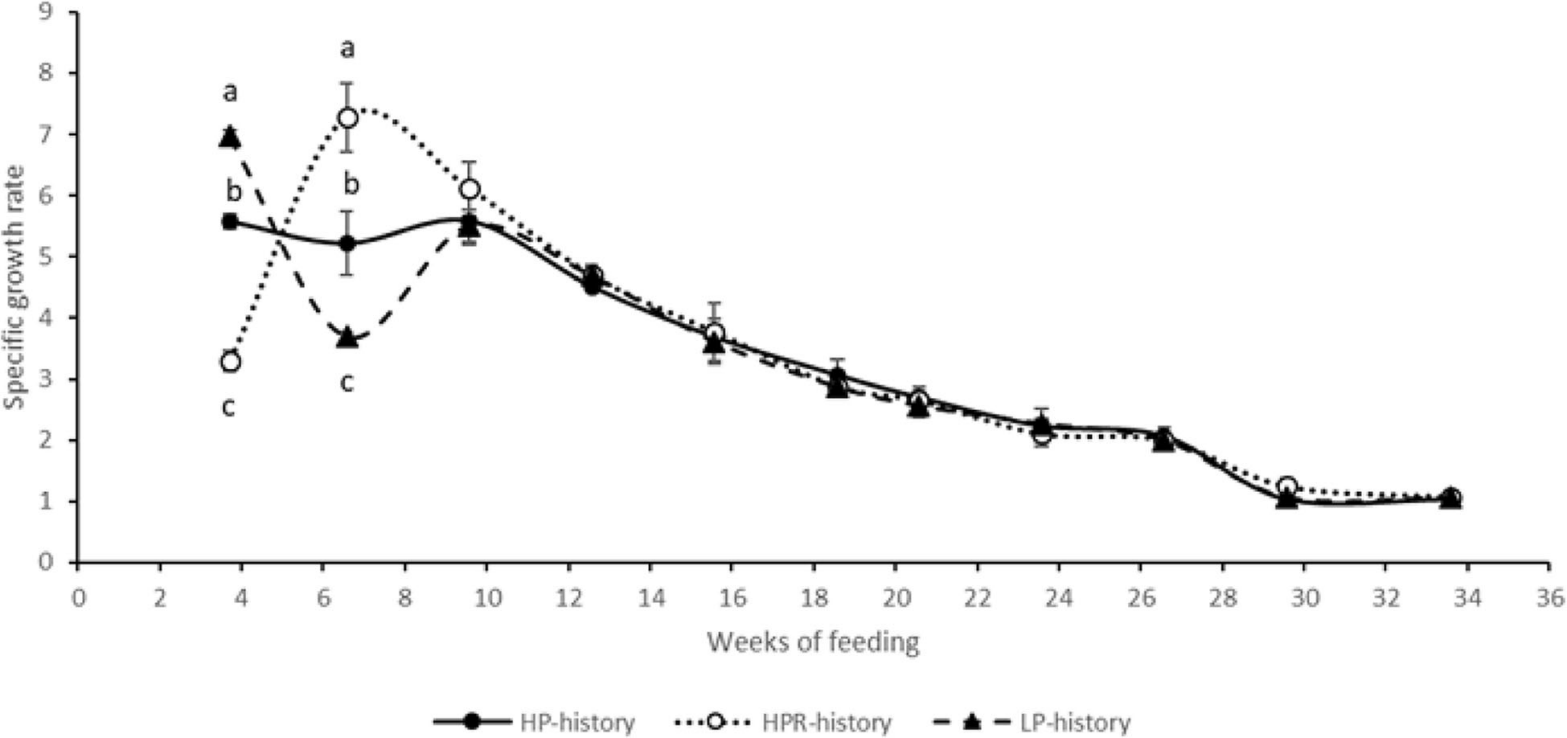
Specific growth rates of the fish from HP, HPR and LP history. HP diet: without carbohydrates. LP diet: with carbohydrates. HPR: fish fed HP diet every other day

Survival and growth performance of fish during the 30-week growth trial are shown in Table 3. No significant difference in survival was observed between the three treatments at the end of stimulus, after the 5–19 weeks of growth trial with commercial diet and after the last dietary challenge test (LP diet). Regarding the growth performance, from 0 to 4 weeks (stimulus period), fish fed LP diet had significantly higher final body mass, specific growth rate and feed efficiency than those fed HP diet (*p* < 0.001, Anova test); fish fed HPR diet had significantly lower final body mass, specific growth rate and feed efficiency than those fed HP diet (*p* < 0.001, Anova test). From 5 to 19 weeks of growth trial (with commercial diet), specific growth rate of fish subjected to LP diet was significantly lower than those fed HP diet whereas specific growth rate of fish in HPR group was significantly higher than those in HP group (*p* < 0.001, Anova test). This was mainly due to the significant increase of specific growth rate just after the stimulus period for the HPR fish (Fig. 2), a well-known process in fish called the compensatory growth [33]. By contrast, after the LP stimulus, fish decreased significantly their specific growth rate when fed with a commercial diet (Fig. 2**)**. After the 20 days, all the fish groups presented the same specific growth rate. Moreover, at the end of the growth trial (commercial diet), i.e. just before the LP challenge, there was no more differences in fish weight due to higher SGR in fish with HP and HPR history. Finally, from 20 to 30 weeks of the growth trial with the LP challenge diet, there was no significant differences in all of the zootechnical parameters measured among the 3 different groups (*p* > 0.05, Anova test).

**Table 3:** Growth performance of rainbow trout during the complete growth trial: A) direct effects of the HP, LP and HPR diets; B) effects of the HP, LP and HPR histories. Data represent means ± SD (*n* = 9 samples per group). Values with different superscripts in the same row are significantly different (*P* < 0.05)

Parameters	Diets			*p-value*
-A -	HP	LP	HPR	
The growth trial 0–4 weeks (stimulus)				
Survival (%)	93.10 ± 0.82	90.95 ± 0.82	90.48 ± 2.06	0.118
Final body weight (g)	0.47 ± 0.01^b^	0.67 ± 0.02^c^	0.26 ± 0.01^a^	<0.001
SGR (% day^−1^)	5.58 ± 0.11^b^	6.97 ± 0.11^c^	3.30 ± 0.16^a^	<0.001
Feed intake (% day^−1^)	3.70 ± 0.35	3.46 ± 0.16	3.15 ± 0.18	0.087
Feed efficiency	1.37 ± 0.14^b^	1.71 ± 0.09^c^	1.05 ± 0.11^a^	0.001
-B-	HP history	LP history	HPR history	
The growth trial 5–19 weeks (commercial diet)				
Survival (%)	99.62 ± 0.66	98.69 ± 0.98	86.15 ± 22.30	0.415
Initial body weight (g)	0.47 ± 0.01^b^	0.67 ± 0.02^c^	0.26 ± 0.01^a^	<0.001
Final body weight (g)	45.33 ± 1.01	47.25 ± 2.64	42.75 ± 2.46	0.111
SGR (% day^−1^)	4.40 ± 0.01^b^	4.09 ± 0.03^a^	4.91 ± 0.08^c^	<0.001
Feed intake (% day^−1^)	2.29 ± 0.06	2.22 ± 0.06	2.68 ± 0.74	0.419
Feed efficiency	1.18 ± 0.03	1.17 ± 0.02	1.22 ± 0.09	0.460
The growth trial 20–30 weeks (dietary challenge with LP diet)				
Survival (%)	98.33 ± 1.67	99.44 ± 0.96	98.89 ± 1.92	0.702
Initial body weight (g)	45.33 ± 1.01	47.25 ± 2.64	42.75 ± 2.46	0.111
Final body weight (g)	196.57 ± 9.48	211.84 ± 7.88	195.75 ± 14.95	0.220
SGR (% day^−1^)	1.90 ± 0.08	1.95 ± 0.09	1.97 ± 0.09	0.621
Feed intake (% day^−1^)	2.31 ± 0.09	2.19 ± 0.06	2.22 ± 0.12	0.334
Feed efficiency	0.93 ± 0.05	0.95 ± 0.06	0.96 ± 0.02	0.747

### Whole body composition and plasma metabolites at the end of the 11-week LP challenge test in rainbow trout juveniles

Whole body composition (crude protein content, crude lipid content and gross energy) and major plasma metabolites (glucose, triglycerides, free amino acids, free fatty acids and lactate levels) were measured in juveniles at the end of the 11-week challenge test (6 h after the last meal). As expected, the glycemia (around 7–8 mM) was higher than usual value (around 5 mM) due to the intake of the carbohydrate-rich LP diet during the challenge period. As shown in Table 4 and Table 5, there was no significant difference among the three groups (HP, LP and HPR dietary histories) in whole body composition parameters and in plasma metabolites levels at the end of the LP challenge test in rainbow trout juveniles (*p* > 0.05, Anova test).

**Table 4:** The effects of HP, LP and HPR diets at the first feeding on whole body composition of juvenile rainbow trout at the end of dietary challenge test. Data represent means ± SD (n = 9 samples per group). Values with different superscripts in the same row are significantly different (*P* < 0.05)

Whole body composition	HP history	LP history	HPR history	*p-value*
Crude protein (%)	16.02 ± 0.14	16.12 ± 0.39	16.32 ± 0.28	0.215
Crude lipid (%)	14.84 ± 0.43	14.26 ± 0.87	14.99 ± 1.82	0.551
Gross energy (kJ g^−1^)	6.91 ± 0.52	6.83 ± 0.40	7.04 ± 0.12	0.661

**Table 5:** The effects of HP, LP and HPR diets at the first feeding on glucose, triglycerides, free amino acids and free fatty acids in the plasma of juvenile rainbow trout at the end of the challenge. Data represent means ± SD (n = 9 samples per group). Values with different superscripts in the same row are significantly different (*P* < 0.05)

Plasma metabolite(mmol/L)	HP history	LP history	HPR history	*p-value*
Glucose	8.28 ± 1.99	7.80 ± 2.22	7.09 ± 1.06	0.396
Triglycerides	3.36 ± 1.04	3.98 ± 1.17	3.58 ± 1.08	0.482
Free amino acids	14.32 ± 1.70	14.79 ± 3.48	12.41 ± 2.32	0.144
Free fatty acids	0.31 ± 0.09	0.32 ± 0.08	0.23 ± 0.05	0.051
Lactate	6.85 ± 0.8	6.55 ± 1.4	7.02 ± 1.7	0.836

### Metabolic gene expressions in rainbow trout alevins at the end of the early stimulus

As shown in Table 6, the mRNA levels of glucose transport-related gene *glut2a*, glycolysis-related genes *gcka*, *gckb*, *pfkla* and *pfkmba* (*p* < 0.01, Anova test) and pyruvate conversion-related genes *ldhaa* and *ldhab* (*p* < 0.001, Anova test) as well as gluconeogenesis-related gene *fbp1a* (*p* < 0.01, Anova test) were significantly higher in alevins which fed the LP diet at first feeding compared with those fed HP diet. Moreover, alevins fed HPR diet at the first feeding had significantly higher mRNA level of gluconeogenesis-related gene *pck1* than those fed HP diet (*p* < 0.02, Anova test). All these observations demonstrated that the stimulus was effectively perceived by trout alevins and translated into molecular reponses.

**Table 6:** The direct effects of HP, LP and HPR diets (at first feeding) on gene expressions of rainbow trout alevins (whole body) for proteins involved in glucose metabolism at the end of the challenge. Data represent means ± SD (n = 9 samples per group). Values with different superscripts in the same row are significantly different (*P* < 0.05)

Target gene	HP diet	LP diet	HPR diet	*p-value*
Glucose transport				
*glut2a*	0.53 ± 0.21^a^	1.20 ± 0.16^b^	0.42 ± 0.26^a^	<0.001
*glut2b*	0.71 ± 0.19	0.85 ± 0.24	0.60 ± 0.21	0.056
*glut4a*	0.90 ± 0.15	0.99 ± 0.20	0.95 ± 0.42	0.778
*glut4b*	0.94 ± 0.21	1.13 ± 0.18	0.91 ± 0.48	0.321
Glycolysis				
*hk1*	0.88 ± 0.27	0.73 ± 0.13	1.13 ± 0.51	0.060
*hk2*	1.38 ± 0.37	1.17 ± 0.24	1.47 ± 0.67	0.386
*pkmaa*	0.80 ± 0.37	0.85 ± 0.21	1.08 ± 0.38	0.192
*pkmab*	0.96 ± 0.17^ab^	1.10 ± 0.14^b^	0.81 ± 0.25^a^	0.015
*pkmba*	0.83 ± 0.13^ab^	0.97 ± 0.20^b^	0.69 ± 0.24^a^	0.023
*pkmbb*	0.75 ± 0.15	0.87 ± 0.23	0.76 ± 0.39	0.601
*gcka*	0.09 ± 0.09^a^	2.94 ± 1.42^b^	0.03 ± 0.01^a^	<0.001
*gckb*	0.04 ± 0.07^a^	2.81 ± 1.10^b^	0.01 ± 0.01^a^	<0.001
*pfkla*	1.06 ± 0.30^a^	1.52 ± 0.47^b^	0.77 ± 0.27^a^	<0.001
*pfklb*	0.99 ± 0.21^ab^	1.20 ± 0.34^b^	0.72 ± 0.24^a^	0.003
*pfkmaa*	1.05 ± 0.60	1.27 ± 0.31	0.87 ± 0.29	0.148
*pfkmab*	1.17 ± 0.59	1.27 ± 0.13	0.81 ± 0.37	0.060
*pfkmba*	0.29 ± 0.25^a^	2.77 ± 3.19^b^	0.10 ± 0.09^a^	0.009
*pfkmbb*	1.08 ± 0.47	1.10 ± 0.13	0.73 ± 0.27	0.038
*pklr*	1.07 ± 0.41^ab^	1.40 ± 0.39^b^	0.78 ± 0.28^a^	0.006
Gluconeogenesis				
*pck1*	0.57 ± 0.45^a^	0.82 ± 0.37^ab^	1.39 ± 0.74^b^	0.012
*pck2*	0.67 ± 0.71	1.42 ± 0.65	0.79 ± 0.62	0.053
*fbp1b1*	1.06 ± 0.31	0.97 ± 0.20	1.03 ± 0.46	0.834
*fbp1b2*	0.97 ± 0.25	1.02 ± 0.25	0.98 ± 0.43	0.946
*fbp1a*	0.95 ± 0.19^a^	1.23 ± 0.21^b^	0.83 ± 0.29^a^	0.004
*g6pca*	1.12 ± 0.41	0.78 ± 0.23	1.01 ± 0.33	0.105
*g6pcb1*	0.87 ± 0.30	1.07 ± 0.26	0.98 ± 0.47	0.486
*g6pcb2*	1.02 ± 0.47	0.96 ± 0.59	1.30 ± 0.68	0.449
Pyruvate conversion				
*ldhaa*	0.93 ± 0.31^a^	1.86 ± 0.60^b^	0.50 ± 0.26^a^	<0.001
*ldhab*	0.83 ± 0.27^a^	1.91 ± 0.61^b^	0.54 ± 0.31^a^	<0.001
Lactate transport				
*slc16a3a*	1.24 ± 0.49	1.04 ± 0.58	0.88 ± 0.64	0.412
*slc16a3b*	1.19 ± 0.29^ab^	0.83 ± 0.20^a^	1.52 ± 0.63^b^	0.007

### Metabolic gene expressions in liver and muscle of rainbow trout juveniles at the end of the 11-weeks LP challenge test

Long-term adaptive changes in gene expression patterns are one of the extremely important biological mechanisms that can be at the origin of a programming effect [1]. We thus analysed mRNA levels for glucose metabolic genes in liver and muscle of juveniles fish challenged with LP diet after early LP and HPR stimuli. As shown in Table 7, there was no significant effect among the three groups on mRNA levels of the metabolic genes involved in glucose metabolism in trout liver. By contrast, significant higher mRNA levels of glucose transport-related gene *glut4a* and glycolysis-related genes *hk2* and *pkmab* were observed in muscle of juvenile fish with LP dietary history compared to those with the HP dietary history (Table 8).

**Table 7:** The effects of HP, LP and HPR diet histories on hepatic gene expressions of juvenile rainbow trout fed LP diet (challenge) for proteins involved in glucose metabolism. Data represent means ± SD (n = 9 samples per group). Values with different superscripts in the same row are significantly different (*P* < 0.05)

Target genes	HP history	LP history	HPR history	*p-value*
Glucose transport				
*glut2a*	1.07 ± 0.19	1.00 ± 0.20	1.07 ± 0.21	0.682
*glut2b*	1.01 ± 0.10	1.00 ± 0.24	1.13 ± 0.25	0.392
Glycolysis				
*gcka*	0.93 ± 0.66	0.97 ± 0.57	0.96 ± 0.37	0.991
*gckb*	1.07 ± 0.43	1.11 ± 0.28	0.98 ± 0.31	0.736
*pfkla*	1.15 ± 0.43	1.09 ± 0.27	1.03 ± 0.25	0.771
*pfklb*	1.09 ± 0.41	1.05 ± 0.25	1.20 ± 0.49	0.719
*pklr*	1.19 ± 0.29	1.14 ± 0.23	1.10 ± 0.18	0.761
Gluconeogenesis				
*pck1*	1.08 ± 0.40	1.08 ± 0.29	0.93 ± 0.33	0.559
*pck2*	1.22 ± 1.03	1.19 ± 0.64	0.75 ± 0.74	0.410
*fbp1b1*	1.33 ± 0.96	1.28 ± 0.50	0.83 ± 0.34	0.213
*fbp1b2*	0.81 ± 0.37	0.94 ± 0.58	1.19 ± 0.60	0.344
*fbp1a*	1.07 ± 0.50	1.05 ± 0.33	1.04 ± 0.27	0.983
*g6pca*	0.99 ± 0.20	1.11 ± 0.28	0.96 ± 0.29	0.455
*g6pcb1*	1.01 ± 0.52	1.35 ± 0.62	0.91 ± 0.39	0.192
*g6pcb2*	1.08 ± 1.08	1.15 ± 0.95	0.97 ± 0.94	0.927
Pyruvate conversion				
*ldhaa*	0.91 ± 0.35	1.18 ± 0.38	1.08 ± 0.33	0.322
*ldhab*	0.99 ± 0.39	0.86 ± 0.22	1.06 ± 0.25	0.346
Lactate transport				
*slc16a3b*	1.21 ± 1.19	2.28 ± 3.19	0.95 ± 0.70	0.359

**Table 8:** The effects of HP, LP and HPR diet histories on muscle gene expressions of juvenile rainbow trout fed LP diet (challenge) for proteins involved in glucose metabolism. Data represent means ± SD (*n* = 9 samples per group). Values with different superscripts in the same row are significantly different (*P* < 0.05)

Target gene	HP history	LP history	HPR history	*p-value*
Glucose transport				
*glut4a*	0.80 ± 0.17^a^	1.84 ± 1.28^b^	0.46 ± 0.14^a^	0.005
*glut4b*	1.13 ± 0.37	0.88 ± 0.28	0.92 ± 0.23	0.191
Glycolysis				
*hk1*	0.95 ± 0.21	0.90 ± 0.28	1.01 ± 0.19	0.612
*hk2*	0.88 ± 0.21^a^	1.35 ± 0.61^b^	0.96 ± 0.27^ab^	0.044
*pkmaa*	1.01 ± 0.33^ab^	1.38 ± 0.64^b^	0.82 ± 0.25^a^	0.036
*pkmab*	0.88 ± 0.14^a^	1.70 ± 1.14^b^	0.66 ± 0.24^a^	0.009
*pkmba*	0.85 ± 0.14	0.97 ± 0.30	0.94 ± 0.45	0.731
*pkmbb*	1.12 ± 0.31	0.85 ± 0.39	1.01 ± 0.42	0.343
*pfkmaa*	0.96 ± 0.29	0.97 ± 0.23	1.03 ± 0.31	0.890
*pfkmab*	1.13 ± 0.26	1.10 ± 0.57	1.04 ± 0.36	0.903
*pfkmba*	1.07 ± 0.42	0.96 ± 0.45	1.15 ± 0.49	0.665
*pfkmbb*	1.13 ± 0.31	1.01 ± 0.39	0.94 ± 0.31	0.509
Pyruvate conversion				
*ldhaa*	1.05 ± 0.39	1.04 ± 0.35	0.95 ± 0.36	0.820
*ldhab*	0.92 ± 0.29	1.08 ± 0.37	0.87 ± 0.33	0.368
Lactate transport				
*slc16a3a*	0.96 ± 0.73	1.40 ± 1.19	0.72 ± 0.57	0.257
*slc16a3b*	1.15 ± 1.28	0.83 ± 0.64	1.05 ± 0.68	0.745

### Glucose enzymes and glycogen in muscle of rainbow trout juveniles at the end of the 11-weeks LP challenge test

**Fig. 3:**
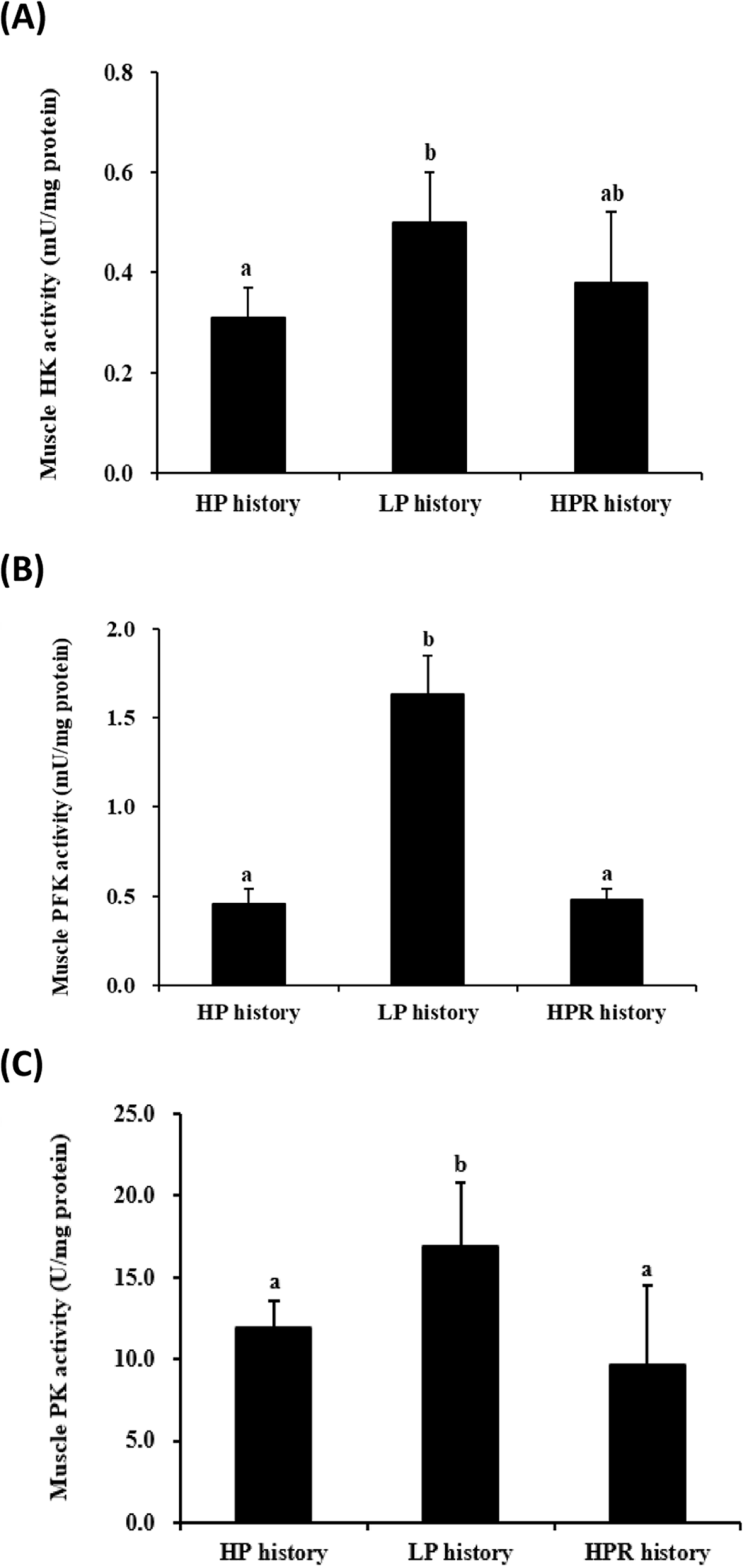
Enzymatic activities of muscle glycolytic enzymes measured in juvenile trout at the end of the LP challenge. **a**. Hexokinase (HK) activity in the muscle of rainbow trout juveniles fed with HP, LP and HPR diets at the first feeding (*p* < 0.02, Anova test). **b.** Phosphofructokinase (PFK) activity in the muscle of rainbow trout juveniles fed with HP, LP and HPR diets at the first feeding (*p* = 3 10^−10^, Anova test). **c.** Pyruvate kinase (PK) activity in the muscle of rainbow trout juveniles fed with HP, LP and HPR diets at the first feeding (*p* = 0.001, Anova test). For all the activities, data were presented as mean ± SD (*n* = 6), Values with different letters are significantly different (*p* < 0.05). HP diet: without carbohydrates. LP diet: with carbohydrates. HPR: fish fed HP diet every other day

**Fig. 4:**
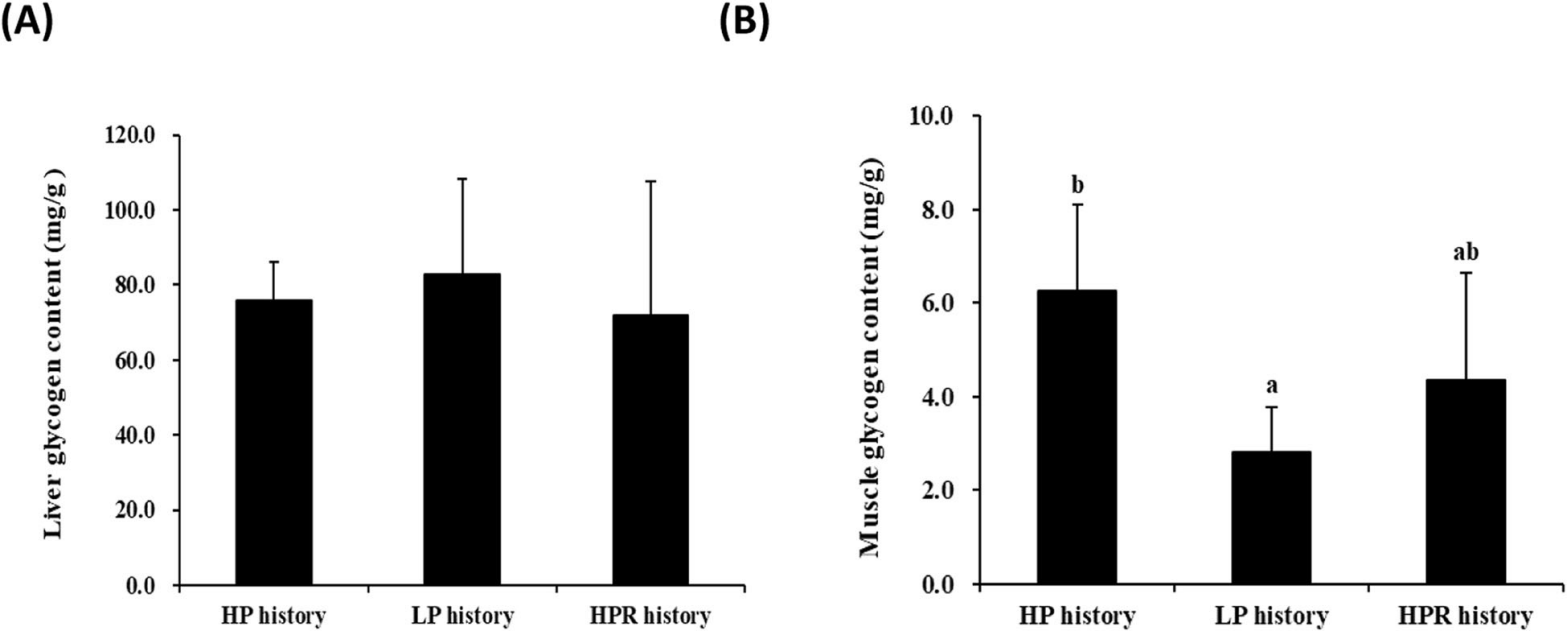
Glycogen contents measured at the end of the LP challenge in the liver (**a**) and muscle (**b**) of rainbow trout juveniles fed with HP, LP and HPR diets at the first feeding (*p*_liver_ = 0.662, *p*_muscle_ = 0.002; Anova test). Data were presented as mean ± SD (*n* = 6), Values with different letters are significantly different (*p* < 0.05). HP diet: without carbohydrates. LP diet: with carbohydrates. HPR: fish fed HP diet every other day

In order to confirm the programming of the glucose metabolism in muscle observed at the molecular level, we analyzed the enzymatic activities of the 3 key glycolytic enzymes. As shown in Fig. 3a, b, c, higher hexokinase, phosphofructokinase and pyruvate kinase activities were noticed in the muscle of fish with LP dietary history compared to those with HP dietary history (*p* < 0.02, Anova test). Moreover, whereas there was no significant effect in the liver (Fig. 4a), there was a significant lower level of glycogen in the muscle of fish with LP dietary history compared to those with HP dietary history (*p* < 0.01, Anova test) (Fig. 4b).

### Global DNA C^m^CGG methylation level in the muscle of juvenile trout at the end of the 11-week LP challenge test

The modification of glucose metabolism (gene expression and activities of the glycolytic enzymes in muscle) associated to the early LP diet intake could be due to an epigenetic mechanism, especially to the level of the DNA C^m^CGG methylation status. We thus estimated the level of such methylation by analysis C^m^CGG content by LUMA assay. As shown in Table 9, global DNA C^m^CGG methylation level in the muscle of trout juveniles with LP dietary history was significantly lower than those with HP dietary history (*p* < 0.01, Anova test).

**Table 9:** The effects of HP, LP and HPR diets at the first feeding on global DNA C^m^CGG methylation level (%C^m^CGG) in the muscle of trout juvenile subjected to a 11-week LP challenge test

Tissue	HP history	LP history	HPR history	*p-value*
Muscle	0.81 ± 0.01^b^	0.79 ± 0.02^a^	0.81 ± 0.02^b^	0.007

## Discussion

Studies about nutritional programming in fish revealed that nutritional stimulus applied at critical developmental stages early in life had persistent effects on physiological/metabolic functions of the organism later in life [10]. Based on the concept of nutritional programming [1–3], the purpose of the present study was to investigate the effects on glucose metabolism of early nutritional stimuli in rainbow trout juveniles using HP, LP and HPR feeding conditions in alevins. Their putative programming effects were tested in juvenile rainbow trout based on growth performance, plasma metabolites, mRNA levels for genes encoding proteins involved in glucose metabolism as well as global epigenetic modification (global DNA C^m^CGG methylation).

### Early stimuli using LP diet and HP restriction feeding have been effective in rainbow trout alevins without decrease of survival

In the present study, higher growth performances (final body mass, specific growth rate and feed efficiency) were noticed in alevins fed LP diet compared with those fed HP diet at the first feeding, suggesting that LP diet at the first feeding could lead to a positive stimulus effect on growth performance in trout alevins. The positive effect of early LP dietary stimulus on growth performance is probably related to the higher lipid content in LP diet compared to the HP diet (16% versus 9.5% respectively) because it is well known that trout alevins efficiently use dietary lipids to support high levels of growth performance [34]. On the other hand, lower growth performances (final body mass, specific growth rate and feed efficiency) were observed in alevins fed HPR diet at the first feeding compared with those fed the HP diet, indicating - as expected - that early dietary restriction (they are fed two times less) caused strong negative impacts on growth performance in trout alevins in the short term.

At the molecular level, results of this study showed that LP dietary stimulus significantly influenced the mRNA levels of some glucose metabolic genes in trout alevins at the end of the first feeding trial. Indeed, the increase in mRNA levels of glucose transporter gene (*glut2a*), glycolysis genes (*gcka*, *gckb*, *pfkla* and *pfkmba*) and pyruvate conversion genes (*ldhaa* and *ldhab*) were found in trout alevins subjected to LP diet compared to those fed HP diet. These data indicated that intake of LP diet, rich in carbohydrates (almost 30%), at the first feeding had expected effect on glycolysis in trout alevins as previously shown in juveniles [35]. However, the increase of one gluconeogenic gene (*fpb1a*) was also observed in LP fish, which was not expected. This phenomenon may be due to the non-inhibition of gluconeogenesis when trout alevins are fed with high carbohydrate diet as observed in our previous studies [36, 37]. On the other hand, in trout alevins following the dietary restriction protocol (HPR), there was only one gene differentially expressed (the gluconeogenic *pck1*) compared to those fed HP diet. Although this result suggested that HPR diet at the first feeding results in an expected up-regulation of the first step of the gluconeogenesis in trout alevins, as previously observed during the fasting stage in alevins [20], the number of glucose metabolic genes modified by the dietary restriction protocol was quite low.

All together, these observations (growth performance and molecular data) indicated that both LP and HPR dietary stimuli were well received by trout alevins, especially at the glucose metabolism level. As no differences in survival were observed at the end of the stimuli, these alevins can thus be used to test the existence of a glucose metabolic programming in juveniles.

### Growth performance and glucose metabolism of rainbow trout juveniles fed LP diet (challenge) were not affected by the early dietary HPR history

In this study, the mRNA levels of glucose metabolism-related genes and glycogen content in the liver and muscle were not influenced by the HPR dietary history, suggesting that using the concept of nutritional programming with early energy restriction to improve dietary carbohydrate utilization in rainbow trout is ineffective to program glucose metabolism. The absence of effects on growth performance, plasma metabolites and whole body composition confirmed the molecular data. We can compare with caution the HPR experimental groups with global caloric restriction models in mammals [3–5]. Moderate maternal caloric restriction programs obesity and even fatty liver in mammals; this was not the case in our fish model, suggesting that the caloric restriction effects at long term is highly dependent of the animal species and maybe linked to the general level of fasting resistance of the species. Indeed, many fish species can tolerate a longer period of prolonged caloric restriction than mammals [38]. For instance, the rates of body mass loss per day in many starving fish (such as rainbow trout, Tilapia and carp) were lower than those in mammals (such as rat, pig and goat) [39–44].

### Glucose metabolism in muscle of rainbow trout juveniles fed LP diet (challenge) was largely modified by the early dietary LP history

Regarding the effects of early LP diet, no significant effect of early dietary LP stimulus at first feeding was found for the whole body biochemical composition (proteins, lipids, energy) in juvenile trout. In the same way, no significant variations of the plasmatic parameters (glycemia, lactate, triglycerides) were found between the experimental groups. Finally, during the challenge trial, we did not observed any significant effect of the diet used at first feeding on the growth performance of juvenile trout. In comparison, Liu et al. [26] reported a negative effect of a 5-days dietary 60% carbohydrates stimulus at first feeding on growth performance of juveniles trout at the end of 24-weeks growth trial whereas Geurden et al. [13, 14] did not observe any difference in growth performances in trout fed a 60% carbohydrates diet for 3 and 5 days at first feeding. It seems that the long-term effect of dietary carbohydrates stimulus on growth performance is highly dependent either of the duration or of the levels of dietary carbohydrates-proteins-lipids ratios of the stimulus.

Besides, the absence of effects on liver has been detected is in accordance with the studies performed by Geurden et al. [14] and Hu et al. [9] using rainbow trout fed high carbohydrate diet (60%) at the first feeding. By contrast, dietary LP stimulus at the first feeding had permanent effect on glucose metabolism in the muscle of juvenile trout. Indeed, higher mRNA levels of muscular glucose transport-related gene *glut4a* was detected in juvenile fish with the LP dietary history compared with those with HP dietary history, suggesting that trout may adapt glucose transport in muscle. Moreover, higher mRNA levels of muscular glycolysis-related genes *hk2* and *pkmab* were observed in juvenile fish with the LP dietary history compared with those with HP dietary history. Meanwhile, the activities of hexokinase and pyruvate kinase in the muscle of fish subjected to LP diet at the first feeding were also significantly higher than those fed HP diet. These above data indicated that dietary LP stimulus at first feeding can strongly regulate hexokinase and pyruvate kinase at mRNA transcript and enzymatic levels in the muscle of trout juveniles. In addition, even though the mRNA levels of phosphofructokinase genes (*pfkmaa*, *pfkmab*, *pfkmba* and *pfkmbb*) in the muscle were differentially expressed among the 3 dietary histories, fish with LP dietary history displayed significantly higher phosphofructokinase enzyme activity in the muscle compared to HP diet control. This indicated that LP dietary history group can only strongly regulate phosphofructokinase at the enzymatic level in the muscle of trout juveniles. Muscle tissue constitutes about 60% of the fish body [45], and so is very important for glucose use in fish [35]. Moreover, enzymatic activities for the glycolytic enzymes in fish with the LP history were also higher compared to fish with the HPR history. In our study, muscular glycogen content in the muscle of fish with LP history was also different i.e. lower level than those with the HP dietary history. Thus, we can associate this lower level of glycogen (storage of excess glucose) to the higher activities of glycolytic enzymes in LP-history fish. Together, all these data indicated that early LP dietary history could, unambiguously, induce higher activities and expression of actors involved in glucose utilization in the muscle of trout juveniles. In fish fed with 60% of carbohydrates (and very low level of proteins 20%) as early stimulus in our previous study [14], we found reverse data i.e. a down regulation of *glut4*, *hk* and *pk* gene expression, suggesting that the programming of glucose metabolism in muscle is strongly dependent of the type of the stimulus (dietary carbohydrate/protein ratio for example). Indeed, in mammals [3–5], a low protein diet in early life is also associated with low levels of *glut4* mRNA and problem of glucose tolerance. As a whole, our findings strongly suggest that, when the levels of proteins is above the requirement (> 35% proteins), the dietary carbohydrates can have a positive effect on glucose metabolism. However, we cannot also eliminate the idea that the level of dietary lipids (different between HP and LP diets) played a role in the muscle programming.

### Level of global C^m^CGG DNA methylation in muscle of rainbow trout juveniles fed LP diet (challenge) was lower in fish with the early dietary LP history

Epigenetic modification can be an important mechanism involved in the long-term metabolic adaptations by early-life environmental stimuli [46]. Global DNA methylation is one of the main markers used for reconstructing the epigenetic state of the genome [47]. In the present study, global DNA C^m^CGG methylation level in the muscle of juvenile trout with LP dietary history was lower than in those with HP and HPR dietary histories, suggesting that this stimulus induced a modification of the genomic stability and may be involved in the modifications of glucose metabolism-related gene expressions observed in this fish. A global DNA hypomethylation (at 5-methylcytosine level) associated with an intake of carbohydrate-rich diet was noted in the previous study performed by Marandel et al. [48] with juvenile rainbow trout. Moreover, DNA hypomethylation was also been documented in metabolic diseases, such as diabetes [49, 50]. Our data clearly showed that a variation of at least one epigenetic mark (C^m^CGG DNA methylation) occurred in muscle, which reinforces the fact that nutritional programming can be mediated through epigenetic mechanisms in rainbow trout as shown in mammals [3–5], especially for the skeletal muscle memory [51].

## Conclusion

In summary, our data demonstrate that LP dietary stimulus at first feeding led to the programming of glucose metabolism in the muscle of trout at the juvenile stage. Moreover, early LP stimulus induced global DNA C^m^CGG hypomethylation in the muscle of juvenile trout. This altered global DNA C^m^CGG methylation level may manifest a way through which dietary LP history permanently modified gene expressions in the muscle of trout juveniles. Nevertheless, our result showed also that dietary HPR stimulus at first feeding exerts no programming influence on glucose metabolism in both liver and muscle of trout juveniles (while being not deleterious for long term growth). Finally, these findings are promising for improving nutritional strategies based on early metabolic programming with different compositions of diets. Further researches are needed to focus on the optimization of the programming conditions in order to change the energy metabolism regulation in this carnivorous fish species.

## Data Availability

The datasets used and analyzed during the current study are available from the corresponding author on reasonable request.
